# Stress Component Decoupling Analysis Based on Large Numerical Aperture Objective Lens, an Impractical Approach

**DOI:** 10.3390/ma15134616

**Published:** 2022-06-30

**Authors:** Ying Chang, Donghui Fu, Mingyuan Sun, Saisai He, Wei Qiu

**Affiliations:** 1 Department of Mechanics, School of Mechanical Engineering, Tianjin University, Tianjin 300072, China; ychang@tju.edu.cn (Y.C.); testfu@tju.edu.cn (D.F.); mingyuansun@tju.edu.cn (M.S.); hesaisai@tju.edu.cn (S.H.); 2 Tianjin Key Laboratory of Modern Engineering Mechanics, Tianjin 300072, China

**Keywords:** micro-Raman spectroscopy, stress state, {100} monocrystalline silicon, Raman-stress relationship, experimental errors

## Abstract

Micro Raman spectroscopy is an effective method to quantitatively analyse the internal stress of semiconductor materials and structures. However, the decoupling analysis of the stress components for {100} monocrystalline silicon (c-Si) remains difficult. In the work outlined, physical and simulation experiments were combined to study the influence of the objective lens numerical aperture (NA) on the Raman stress characterization. The physical experiments and simulation experiments show that the spectral results obtained by using lenses with different NAs can accurately obtain the principal stress sum but cannot decouple the components of the in-plane stress. Even if the spectral resolution of the simulated experiment is ideal (The random errors of the polarization directions of less than ±1° and the systematic random errors of less than ±0.02 cm^−1^). The analysis based on the theoretical model demonstrates that the proportion of the principal stress sum in the Raman shift obtained in an actual experiment exceeded 98.7%, while the proportion of the principal stress difference part was almost negligible. This result made it difficult to identify the variable effects of different stress states from the experimental results. Further simulation experiments in this work verify that when the principal stress sum was identical, the differences in the Raman shifts caused by different stress states were much smaller than the resolution of the existing Raman microscope system, which was hardly possible to identify in the experimental results. It was proven that decoupling analysis of stress components using the large-NA objective lens lacked actual practicability.

## 1. Introduction

The development of semiconductor science and information devices has become an essential condition to promote artificial intelligence, electronic communication, automotive electronics, big data centres and other fields. The performance of semiconductor devices is supported by material advancement from traditional semiconductor materials (silicon, germanium) to third-generation semiconductor materials (gallium nitride, silicon carbide) to ultrawide bandgap semiconductor materials (diamond, gallium oxide). This continuously promotes the innovation and development of radio frequency communication, high-power devices, lighting devices, etc. One of the keys that affect the semiconductor device performance is strain engineering [1,2], whose foremost requirement is precise control of stress.

The internal stress in a semiconductor device is spatially unevenly distributed and in a complex stress state due to the complexity of the structure. Studies have shown that the stress state is the dominant factor that affects the device reliability [3] and the crucial factor that affects the electron mobility [4] and quantum spin [5] of semiconductors. The accurate quantitative characterization of the stress or strain state of semiconductors is a vital segment in the design and manufacture of microelectronics and a frontier scientific issue at the intersection of solid-state physics and experimental mechanics.

There are many microscale experimental mechanical methods to characterize stress states, such as X-ray diffraction (XRD), TEM moiré, and micro-Raman spectroscopy. Among them, micro-Raman spectroscopy has achieved many applications in the characterization of internal stresses in low-dimensional materials, the analysis of microdevice processes and residual stresses, and the mechanical behaviour of microscale interfaces due to its advantages of high resolution, nondestructive noncontact, sensitivity to both intrinsic and nonintrinsic stresses. Zhao et al. [6,7,8,9,10] studied stress or strain in fracture characteristics, boundary effects, transfer, interface reinforcement of graphene by Raman spectroscopy. The investigations of Xie et al. [11,12,13,14,15] found that lithium storage behaviour, capacity and lifetime of graphene electrodes lithium-ion (Li^+^) batteries are strongly affected by the microstructure and stress/strain. There are still several bottlenecks that must urgently be solved, although much evolution has been made in terms of its characterization theoretical model, critical experimental techniques, and high-level analytical instruments in recent years. Prominently, the decoupling problem of stress state components for typical materials has become a common and difficult problem in Raman stress analysis.

In the existing research, the basic mentality of stress state analysis using micro-Raman spectroscopy is as follows. Substituting the stress–strain relationship into the lattice dynamics secular equation and solving the relationship between each component of the stress tensor and the Raman shift, the analytical expression of the relationship between the measured Raman shift and the stress component is obtained based on the Raman selection rule. De Wolf derived the stress-Raman shift relationship under uniaxial stress and biaxial stress based on simplification by neglecting the shear stress for c-Si [16]. Ma et al. proved that the backscattering Raman measurement results of {100} c-Si were independent of the shear stress through theoretical derivation and calibration experiments, which is due to the triple degeneracy of the Raman peaks of c-Si. Decoupling analysis of stress components cannot be achieved because the Raman shift is linearly related to the sum of the principal stresses [17,18]. To decouple stress components, Loechelt et al. proposed a method for decoupling analysis using off-axis measurement and developed a related device [19]. Fu et al. used a Raman measurement method based on oblique backscattering to realize the decoupling of in-plane bidirectional stress [20]. In contrast, most applied studies have focused on the inability to decouple measurements by resorting to theoretical models of elasticity [21], finite element analysis [22], simplifying assumptions on stress states [23], or simply ignoring stress states and actually treating stresses as scalars [24].

In recently years, several published works claimed that the stress/strain states of {100} c-Si were or could be decoupled through MRS with large numerical aperture (NA) lenses. Some researchers have found that Raman measurements with large-NA objective lens can activate the forbidden TO mode of {100} c-Si, which obtains more abundant Raman information to help decouple the stress components. Kosemura observed transverse optical phonons of {100} c-Si with a large-NA lens [25]. Brunner et al. determined the biaxial stress at the SiO_2_/Si interface by using a Raman selection rule and a finite solid angle of collection, which can monitor all modes in the [001] crystalline SiO_2_/Si structure [26]. In addition, some other works have been performed using large-NA objectives. However, their stress states are uniaxial or biaxial in fact [26,27,28,29], which is not relevant to the decoupling analysis of complex stress states (regardless of what they claim [30]). Furthermore, for complex silicon samples such as polysilicon and textured silicon [27,31], experiments have shown that a large NA (NA > 0.4) indeed provides more abundant experimental information than a small NA (NA ≤ 0.4) [28,30,32]. However, there has been a lack of a theoretical and experimental basis for the problem of decoupling the analysis of complex stress states with large-NA objective lenses, such as {100} c-Si.

For these issues, some influences were introduced into the Raman-stress characterization model, such as refraction, receding deflection, and large NA, based on the basic theory of experimental mechanical characterization. The analytical relationship between the measured Raman shift and the in-plane stress components was derived for different NA backscattering configurations. The effect of the objective lens NA on the stress characterization in the micro-Raman system was quantitatively verified by a combination of physical and simulation experiments.

## 2. Materials and Experiments

### 2.1. Physical Experiments

Double-polished (100) c-Si was invoked as the sample (Guangzhou Fangdao Semiconductor Co., Ltd., Guangzhou, China). The Raman experiments were performed using a self-developed microscopic Raman device [33], which was scanned and probed in the HV-case (HV-case was defined when the polarization direction of incident laser was perpendicular to that of scattering light, i.e., *γ* = *φ* + 90°) and vertical backscattering configurations using objective lenses with NA = 0.55, NA = 0.7, and NA = 0.8, respectively. The mapping region was 0.9 mm × 1.2 mm in the centre of the sample with a sampling point spacing of 0.3 mm and 20 points in total and repeat once. All experiments had an identical integration time of 3 s, a 532 nm laser output power of 150 mW, a spectrometer grating of 1800 L/mm. In general, laser power should be kept below a few tens of µW to reduce thermal effects [34,35]. To eliminate thermal effects, the relative increment of Raman shift between the state under stress and that of stress-free is used in the experiments.

**Figure 1:**
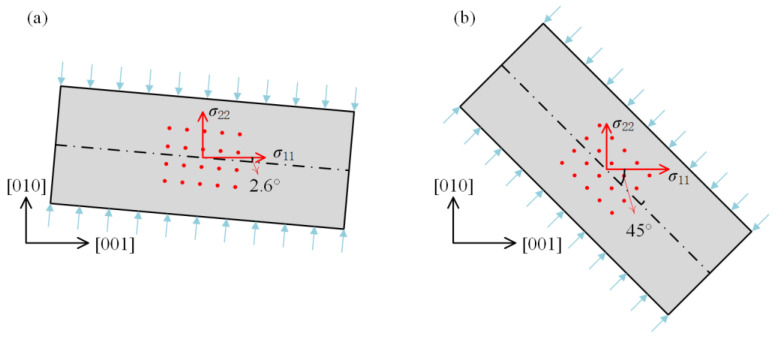
Schematic diagram of the sample loading method: (**a**) sample 1; (**b**) sample 2.

The samples were loaded as shown in Figure 1 with compressive stresses of *σ*_S1_ = −117.84 MPa and *σ*_S2_ = −58.78 MPa for sample 1 and sample 2, respectively. After the calculation, the stress components in the crystal coordinate system are given in Table 1.

### 2.2. Simulation Experiments

Origin (2018, 9.5) was employed for simulation experiments. First, the principal stress difference coefficients were given for six different NAs (NA is 0.28, 0.55, 0.70, 0.80, 0.95, and 1.30) based on the Raman shift-stress relationship considering the NA, and the fraction of the non-principal stress sum was obtained as a percentage of the total Raman shift. Subsequently, two stress states with identical principal stress sum but different stress components, i.e., biaxial (*σ*_11_ = *σ*_22_ = −500 MPa) and unequal biaxial (*σ*_11_ = −700 MPa, *σ*_22_ = −300 MPa), were chosen to give the expected Raman shifts of the angle-resolved Raman spectra at different NAs in steps of 10° of the polarization direction.

## 3. Models and Methods

**Figure 2:**
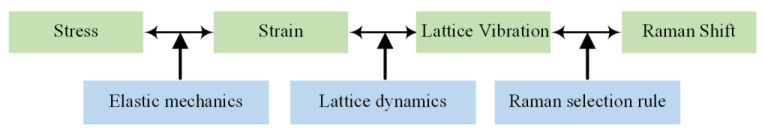
General Raman-stress relationship model building flow chart.

The process of establishing the Raman-stress relationship is shown in Figure 2.

According to the generalized Hooke’s law in elasticity theory, there is a linear relationship between stress and strain in the case of small deformation, and its general form is shown in Equation (1).

(1) ε=Sσ

where ***ε*** is the strain tensor, ***S*** is the elasticity tensor, and ***σ*** is the stress tensor.

The lattice dynamics secular equation for the effect of strain is shown in Equation (2).

(2) $$\begin{array}{l} \left|\begin{array}{ccc} \varepsilon_{uv}K_{uv11}-\lambda & \varepsilon_{uv}K_{uv12} & \varepsilon_{uv}K_{uv13}\\ \varepsilon_{uv}K_{uv21} & \varepsilon_{uv}K_{uv22}-\lambda & \varepsilon_{uv}K_{uv23}\\ \varepsilon_{uv}K_{uv31} & \varepsilon_{uv}K_{uv32} & \varepsilon_{uv}K_{uv33}-\lambda \end{array}\right|=0\\ u,v=1,2,3 \end{array}$$

where *ε_uv_* is the strain component, *K_uvij_* is the phonon deformation potential tensor component, and *K_uvij_ = K_ijuv_ = K_vuij_ = K_uvji_*, *i*, *j* = 1, 2, 3. The eigenvalues *λ_k_* (*k* = 1, 2, 3) and corresponding eigenvectors ***n****_k_* are obtained by solving Equation (2). Let the Raman shifts in the strain-free and strained states of the lattice be *ω*_0_ and *ω_k_*, respectively, *λ_k_* = *ω_k_*^2^ − *ω*_0_^2^. Generally, the difference between *ω*_0_ and *ω_k_* is small, so the Raman shift increment Δ*ω_k_* can be approximated as Equation (3).

(3) $$\Delta \omega_{k}=\omega_{k}-\omega_{0}\approx \frac{\omega_{k}^{2}-\omega_{0}^{2}}{2\omega_{0}}=\frac{\lambda_{k}}{2\omega_{0}}$$

**Figure 3:**
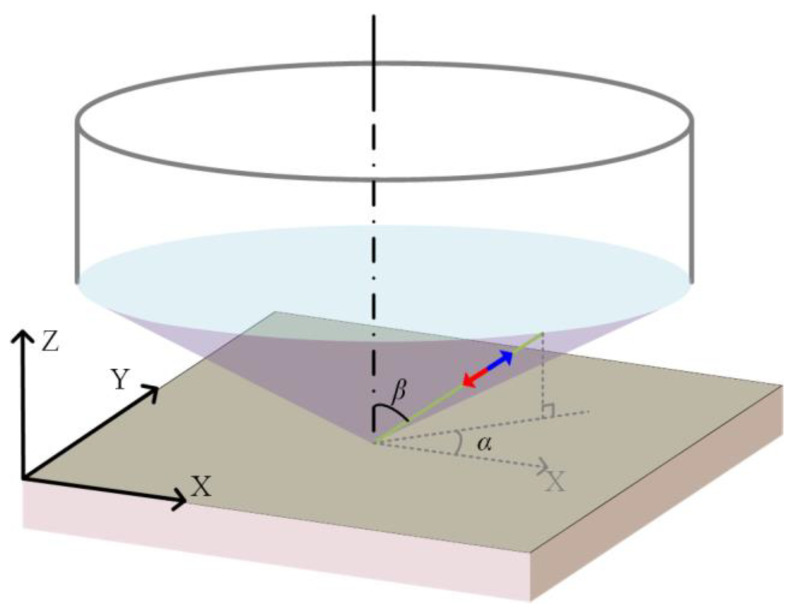
Diagram of the Raman intensity integration with a large-NA objective lens.

In the measured Raman spectral information, the intensity of each characteristic peak *I_l_* is determined by the Raman selection rule, as shown in Equation (4).

(4) $$I_{l}=C{\left|e_{i}^{T}\cdot R_{l}\cdot e_{s}\right|}^{2},l=1,2,3$$

where ***e****_i_* and ***e****_s_* are the polarization vectors of incident laser and scattering light, respectively, and ***R**_l_* is the Raman tensor in the crystal coordinate system. The Raman intensity is actually the sum of the Raman scattering light excited by incident laser in the optic cone [33]. The Raman scattering intensity is integrated in different directions within the optic cone under the microscope (as shown in Figure 3) as shown in Equation (5), where *α* is the angle between the projection of the light in the X-Y plane and the *X*-axis, and *α* ∈ [0, 2π]; *β* is the angle between the light and the lens axis (i.e., *Z*-axis), and *β* ∈ [0, arcsin(NA)]. A general formula for the Raman intensity is given as Equation (5) based on [33] in this paper, where *F_l_* is the integration result of the intensity of each Raman characteristic peak *I_l_*.

(5) $$F_{l}=\int_{0}^{\mathrm{arc}\sin(N.A.)}\int_{0}^{2\pi }I_{l}d\beta d\alpha =C\int_{0}^{\mathrm{arc}\sin(N.A.)}\int_{0}^{2\pi }{\left|e_{i}^{T}\cdot R_{l}\cdot e_{s}\right|}^{2}d\beta d\alpha ,l=1,2,3$$

The relationship between the measured Raman shift Δ*ω_obs_* and the increment of each phonon Raman shift Δ*ω_k_* weighted by their respective contributions to the total scattering intensity is shown in Equation (6).

(6) $$\Delta \omega_{obs}=\frac{\sum_{k=1}^{3}\Delta \omega_{k}F_{k}}{\sum_{k=1}^{3}F_{k}}$$

The Raman shift-stress relationship for the specific NA can be obtained as shown in Equation (7) using Equations (1)–(6), where the stress unit is GPa.

(7) $$\Delta \omega_{obs}=-\frac{\left(1.7127K_{1}+2.3006\right)\left(\sigma_{11}+\sigma_{22}\right)+0.5879K_{2}\left(\sigma_{11}-\sigma_{22}\right)}{K_{1}+1}$$

where,

(8) $$\left\{ \begin{array}{l} K_{1}=a_{1}\cos^{2}\varphi +b_{1}\cos^{2}\gamma +c_{1}\cos\varphi \cos\gamma \cos\left(\gamma -\varphi \right)\\ K_{2}=a_{2}\cos^{2}\varphi +b_{2}\cos^{2}\gamma +c_{2}\cos\varphi \cos\gamma \cos\left(\gamma -\varphi \right) \end{array}\right.$$

In Equation (8), *φ* and *γ* are the polarization directions of incident laser and scattering light, respectively. In this paper, the HH-case was defined when the polarization direction of the incident laser was parallel to that of the scattering light, i.e., *φ* = *γ*. The HV-case was defined when the polarization direction of the incident laser was perpendicular to that of the scattering light, i.e., *γ* = *φ* + 90°. The coefficients *a*_1_, *b*_1_, *c*_1_, *a*_2_, *b*_2_, and *c*_2_ were calculated by integrating Equation (5). These coefficients are given in Table 2 for different values of different NAs.

In the HH-case, parameters *K*_1_ and *K*_2_ became,

(9) $$\begin{array}{l} K_{1}=\left(a_{1}+b_{1}+c_{1}\right)\cos^{2}\varphi \\ K_{2}=\left(a_{2}+b_{2}+c_{2}\right)\cos^{2}\varphi \end{array}$$

In the HV-case, parameters *K*_1_ and *K*_2_ became,

(10) $$\begin{array}{l} K_{1}=a_{1}\cos^{2}\varphi +b_{1}\sin^{2}\varphi \\ K_{2}=\left(a_{2}\cos^{2}\varphi +b_{2}\sin^{2}\varphi \right) \end{array}$$

## 4. Results and Discussion

**Figure 4:**
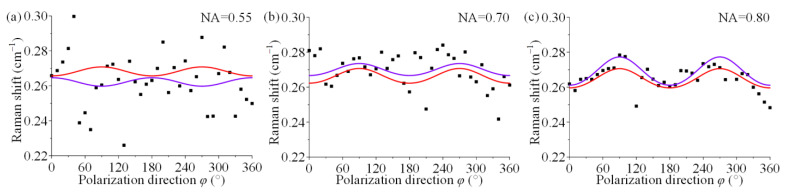
Experimental results of sample 1 with different NAs: (**a**) NA = 0.55, (**b**) NA = 0.70, (**c**) NA = 0.80, where black solid points are experimental data points, the red curve is the theoretical result, and the purple curve is the fitted result.

The physical experimental results for sample 1 at different NAs in Figure 4. The black solid points are the experimentally measured data points, the red curve is the theoretical result for the stress state, and the purple curve is the fitted result using Equation (7). When NA = 0.55, the fitted curve differs from the theoretical curve more, and even the trend is not consistent from Figure 4a. When NA = 0.7 and NA = 0.8, the fitted curves follow the same trend as the theoretical curves but do not completely overlap in Figure 4b,c. The results of fitted stress components for NA = 0.55 are *σ*_11/0.55_ = −1770.36 MPa, *σ*_22/0.55_ = 1657.67 MPa. The results of fitted stress components for NA = 0.70 are *σ*_11/0.70_ = −204.46 MPa, *σ*_22/0.70_ = 85.42 MPa. The results of fitted stress components for NA = 0.8 are *σ*_11/0.80_ = −120.83 MPa, *σ*_22/0.80_ = 0 MPa.

**Figure 5:**
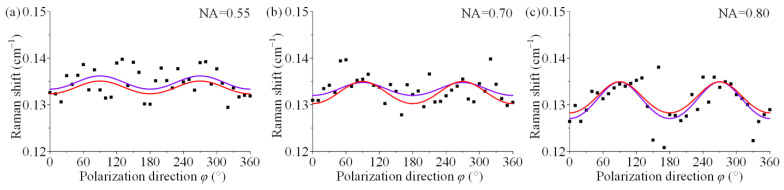
Experimental results of sample 2 with different NAs: (**a**) NA = 0.55, (**b**) NA = 0.70, (**c**) NA = 0.80, where black solid points are experimental data points, the red curve is the theoretical result, and the purple curve is the fitted result.

The physical experimental results were given for sample 2 at different NAs in Figure 5. Again, the black solid points are the experimentally measured data points, the red curve is the theoretical result for the stress state, and the purple curve is the fitted result using Equation (7). The fitted curves at three NAs show that the fitted curves follow the same trend as the theoretical curves, but the curves do not overlap. The results of fitted stress components for NA = 0.55 are *σ*_11/0.55_ = −16.78 MPa, *σ*_22/0.55_ = −42.48 MPa. The results of fitted stress components for NA = 0.70 are *σ*_11/0.70_ = −132.61 MPa, *σ*_22/0.70_ = 73.96 MPa. The results of fitted stress components for NA = 0.8 are *σ*_11/0.80_ = −0.3 MPa, *σ*_22/0.80_ = −58.48 MPa.

Furthermore, the principal stress sum was separately calculated for sample 1 and sample 2 in this paper. The principal stress sums for the experimental results of sample 1 are (*σ*_11_ + *σ*_22_)_0.55_ = −112.69 MPa, (*σ*_11_ + *σ*_22_)_0.70_ = −119.04 MPa, and (*σ*_11_ + *σ*_22_)_0.80_ = −120.83 MPa, respectively. The principal stress sums for the experimental results of sample 2 are (*σ*_11_ + *σ*_22_)_0.55_ = −59.26 MPa, (*σ*_11_ + *σ*_22_)_0.70_ = −58.68 MPa, (*σ*_11_ + *σ*_22_)_0.80_ = −58.78 MPa. The experimental results did not differ much from the sum of the applied principal stresses after the sum of the principal stresses was calculated for these two stress states (as shown in Table 1).

The average relative error of stress components and principal stresses sum were calculated based on the experimental results, as shown in Table 3. The average relative error range of the stress components exceeds 43.73% from Table 3. The average relative error is not related to the value of NA, while all relative errors of the principal stress sum are less than 5% from the error distribution.

The large NA cannot be used for decoupling when observing the results of physical experiments. Specifically, an obvious gap remains between the fitted curves and the theoretical curves in most cases (Figure 4b,c and Figure 5a–c), and the fitted curves cannot agree with the theoretical trend because the experimental points are too scattered in a few cases (Figure 4a). The results of all stress components based on the experimental data are significantly different from the actual applied stress state, and there is no indication that the trend is related to the NA of objective lens, which indicates that the use of large-NA lens does not help to decouple stress components. The calculation of the principal stress sum reveals that the proposed method was used to analyse the angle-resolved Raman data in most cases to more accurately identify the principal stress sum, although the experimental points were far from the theoretical curves. Thus, the fitting method can strongly shield single-point errors [36]. However, the relative error of the principal stress sum also exhibits a weak correlation with NA, so the analytical consequences of the principal stress sum are independent of magnitude of NA.

The ability of backscattering micro-Raman to probe the sum of principal stresses in {100} c-Si is well known. Using a characterization model that considered NA, refraction and depolarization as in this paper, or a generic model that did not consider NA, refraction and depolarization [18,37], or even the classical method by de Wolf that ignored the effect of shear stress and simplified stress state [23], it is possible and only possible to characterize the principal stresses sum of {100} c-Si using backscattering micro-Raman. Some papers had theoretically concluded that the use of a large-NA objective lens could excite and collect TO mode information and “could” contribute to the decoupling of stress components from {100} c-Si [30]. However, experiments on different stress states using a large-NA objective lens in this paper confirm that the difference in stress components hardly affects the Raman shift, and it is difficult to effectively extract the information of stress components from Raman information. It is impossible to obtain the individual stress components in {100} c-Si unless the relationship between the two principal stresses is predicted, regardless of the NA of the objective lens.

*K*_3_ is defined as the ratio of the coefficient in front of the principal stress difference to that of the principal stress sum in Equation (7) to clarify the reason for the weak decoupling effect of NA on stress components.

(11) $$K_{3}=\frac{0.5879K_{2}}{\left(1.7127K_{1}+2.3006\right)}$$

**Figure 6:**
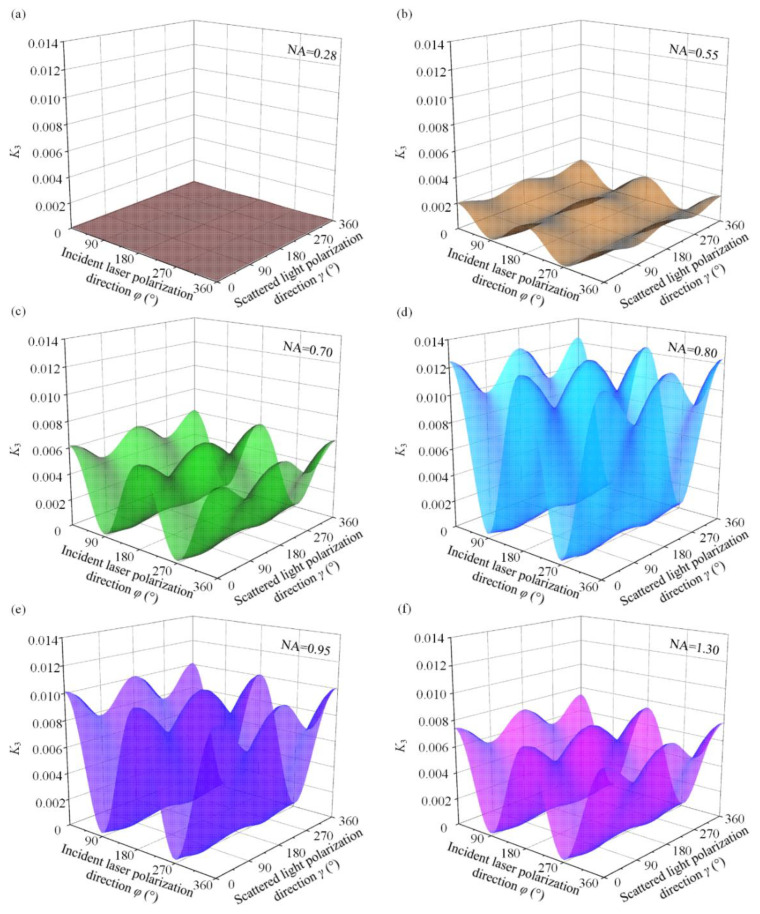
Ratio of the coefficient of the principal stress difference to that of the principal stress sum for different NA (*K*_3_): (**a**) NA = 0.28; (**b**) NA = 0.55; (**c**) NA = 0.70; (**d**) NA = 0.80; (**e**) NA = 0.95; (**f**) NA = 1.30.

**Figure 7:**
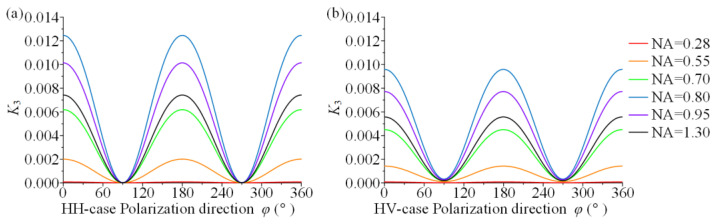
Ratio of the coefficient of the principal stress difference to that of the principal stress sum for different NA (*K*_3_): (**a**) HH-case; (**b**) HV-case.

*K*_3_ was calculated for different NA at different polarization directions and the results (as drawn by Origin in Figure 6) show that the magnitude of NA does correlate with coefficient *K*_3_, but the effect is not monotonic. When NA = 0.80, *K*_3_ is the maximum value, which is 0.01245. These result shows that the *K*_3_ maximum value does not exceed 0.013, which implies that the sum of principal stresses accounts for at least 98.7%, even close to 100%, of the total Raman shift. In other words, the use of large NAs increases the proportion of the principal stress difference in the total Raman shift. However, this proportion is still negligible, which makes it a roadblock to decouple the stress components using a large-NA objective lens. Figure 7 displays the effect of NA on *K*_3_ for two common polarization configurations, i.e., the HH-case and HV-case, which are more visually represented by the curves in these two configurations.

The simulation experimental results of stress decoupling are given for six different NA (NA = 0.28, 0.55, 0.70, 0.80, 0.95, 1.30) based on the Raman shift-stress relationship (Equation (7)) to demonstrate the effect of different NA on the stress analysis. The simulation experimental was obtained by substituted the stress state and polarization configuration (HV-case) into Equation (7) and then drawn the Raman shift-polarization direction curves. The stress states were chosen as biaxial (*σ*_11_ = *σ*_22_ = −500 MPa) and unequal biaxial (*σ*_11_ = −700 MPa, *σ*_22_ = −300 MPa). The two stress states have identical principal stress sums, and the stress components are large (exceeding the compressive strength) and will be difficult to achieve in physical experiments due to sample breakage.

**Figure 8:**
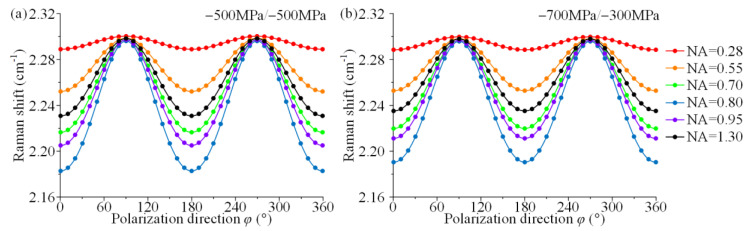
Simulation experiment of (**a**) biaxial (*σ*_11_ = *σ*_22_ = −500 MPa) and (**b**) unequal biaxial (*σ*_11_ = −700 MPa, *σ*_22_ = −300 MPa) stress states at different NA.

The results of the simulation experiments are shown in Figure 8. The solid dots indicate the corresponding Raman shifts in different polarization directions, and the solid lines indicate the fitted curves of the dots. Red, orange, green, blue, purple and black correspond to NAs of 0.28, 0.55, 0.70, 0.80, 0.95 and 1.30, respectively. The polarization direction-Raman shift curves are basically identical for different stress states with identical NAs although the two stress states are different, as shown in Figure 8a,b.

**Figure 9:**
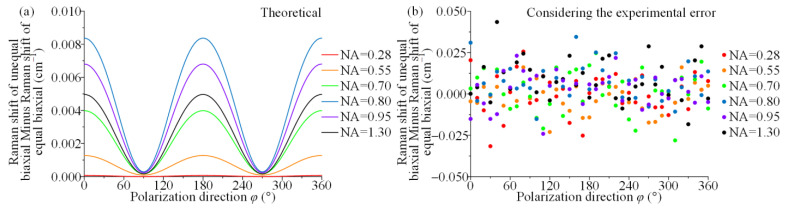
Difference in Raman shifts of the unequal biaxial and biaxial stress states of (**a**) theoretical and (**b**) introducing the random error in polarization direction and system.

The difference of the respective Raman shifts was taken at six different NAs, i.e., the curve of Figure 8b was subtracted from the curve of Figure 8a for identical NAs, and the result is shown in Figure 9a. The maximum value of the difference in Raman shifts does not exceed 0.009 cm^−1^ from Figure 9a. Such a small Raman shift difference occurs at a large magnitude of stress, which is difficult to distinguish for existing Raman spectroscopy instrument systems. In general, advanced research-grade Raman spectroscopy systems can achieve a spectral resolution of 0.1 cm^−1^. Ideally, the spectral repeatability of a c-Si for calibration is up to ±0.015 cm^−1^. The range of systematic random error significantly exceeds the above range due to environmental, operational and other factors in practice. Thus, the difference in Raman shifts due to the difference in stress state is much smaller than the random error of the system, which results in its nonrecognition. Furthermore, the main factors that may cause errors also include random polarization errors in angle-resolved Raman experiments. The random errors of the polarization directions of less than ±1° and the systematic random errors of less than ±0.02 cm^−1^ were introduced in the simulation experiments using the experimental error introduced method by Qiu et al. [38]. The difference in Raman shifts was derived from two different stress states under the same polarization state as in Figure 9b. Figure 9b shows that the distribution of the difference in Raman shifts does not have any pattern, and the maximum is close to 0.05 cm^−1^ after the error is introduced. These haphazard Raman shift differences are not caused by variability with stress state but are caused by introducing two measurement errors. Thus, it is difficult for the current micro-Raman instrument to identify the difference in stress states and decouple the stress components in the case of identical principal stress sums.

Accurate characterization of stresses in c-Si of {100} family of crystal planes is imperative for the semiconductor industry and related research. The work in this paper demonstrates that the use of large-NA objective lens does not contribute to the decoupling analysis of stress components. Fu et al. proposed a method using oblique scattering for stress analysis, which can effectively excite and collect information from the transverse optical phonon mode and achieve in-plane stress component decoupling [20].

## 5. Conclusions

In recently several published works by others claimed that the stress/strain states of {100} c-Si were or could be decoupled through MRS with large numerical aperture (NA) lenses. In this paper, for the stress components decoupled from {100} c-Si, angle-resolved Raman experiments using different NA objectives were compared based on a theoretical model for different stress states by combining physical and simulation experiments. It was proven that decoupling analysis of stress components using any large-NA objective lacked practicability:

1. The backscattering Raman can give a relatively accurate sum of the principal stresses in {100} c-Si, regardless of the NA size. However, decoupling analysis of the in-plane stress components cannot be achieved.
2. This work finds that the principal stress sum is more than 98.7% of the total Raman shift, which is close to 100% by analysing the theoretical model and simulation experiment results. The principal stress difference part, which can represent the variability of stress state, has a subtle weight in the total Raman shift.
3. The Raman shift variation caused by stress state is much smaller than the resolution of existing micro-Raman system, which cannot be identified in the experimental results with the combination of various experimental errors.

These factors ultimately lead to the lack of realistic value of utilizing large-NA objective lens to perform the stress component decoupling analysis.

## Figures and Tables

**Table 1:** Stress components and principal stress sum of sample 1 and sample 2.

	*σ*_11_/MPa	*σ*_22_/MPa	*τ*_12_/MPa	(*σ*_11_ + *σ*_22_)/MPa
Sample 1	−117.60	−0.24	−5.34	−117.84
Sample 2	−29.39	−29.39	−29.39	−58.78

**Table 2:** Coefficients *a*_1_, *b*_1_, *c*_1_, *a*_2_, *b*_2_, and *c*_2_ values for different NAs.

	*a*_1_	*b*_1_	*c*_1_	−*a*_2_	−*b*_2_	−*c*_2_
NA = 0.20	0.0105	0.0006	0.0048	0 *	0 *	0.0004
NA = 0.28	0.0209	0.0011	0.0097	0.0003	0 *	0 *
NA = 0.42	0.0495	0.0026	0.0225	0.0023	0.0009	0.0007
NA = 0.55	0.0909	0.0041	0.0397	0.0059	0.0004	0.0023
NA = 0.70	0.1684	0.0066	0.0663	0.0198	0.0007	0.0080
NA = 0.80	0.2526	0.0084	0.0916	0.0446	0.0012	0.0157
NA = 0.95	0.1957	0.0065	0.0710	0.0346	0.0010	0.0121
NA = 1.30	0.1360	0.0045	0.0493	0.0240	0.0007	0.0084

* indicates a value less than 5 × 10^−5^.

**Table 3:** Average relative errors of stress components and principal stress sums in physical experiments.

	Sample 1		Sample 2	
	Average Relative Error of Stress Components	Relative Error of Principal Stress Sums	Average Relative Error of Stress Components	Relative Error of Principal Stress Sums
NA = 0.55	346,100.00%	4.37%	43.73%	0.82%
NA = 0.70	17,882.77%	1.02%	351.43%	0.17%
NA = 0.80	5001.38%	2.54%	98.98%	0%

## Data Availability

The data that support the findings of this study are available from the corresponding author upon reasonable request.

## References

[1] Parton E., Verheyen P. (2006). Strained silicon—The key to sub-45 nm CMOS. III-Vs Rev..

[2] Chidambaram P.R., Bowen C., Chakravarthi S., Machala C., Wise R. (2006). Fundamentals of silicon material properties for successful exploitation of strain engineering in modern CMOS manufacturing. IEEE Trans. Electron. Dev..

[3] Calabretta M., Sitta A., Oliveri S.M., Sequenzia G. (2020). An Integrated Approach to Optimize Power Device Performances by Means of Stress Engineering. Design Tools and Methods in Industrial Engineering.

[4] Dhar S., Ungersbock E., Kosina H., Grasser T., Selberherr S. (2007). Electron mobility model for (100) stressed silicon including strain-dependent mass. IEEE Trans. Nanotechnol..

[5] Lafuente-Sampietro A., Utsumi H., Boukari H., Kuroda S., Besombes L. (2016). Individual Cr atom in a semiconductor quantum dot: Optical addressability and spin-strain coupling. Phys. Rev. B.

[6] Wang Y.L., Wang Y., Xu C., Zhang X.W., Mei L., Wang M., Xia Y., Zhao P., Wang H.T. (2018). Domain-boundary independency of Raman spectra for strained graphene at strong interfaces. Carbon.

[7] Jin Y., Ren Q., Liu J., Zhang Y., Zheng H., Zhao P. (2022). Stretching graphene to 3.3% strain using formvar-reinforced flexible substrate. Exp. Mech..

[8] Yao Q.Z., Qi Y.Z., Zhang J., Zhang S., Zhao P., Wang H.T., Feng X.Q., Li Q.Y. (2019). Impacts of the substrate stiffness on the anti-wear performance of graphene. AIP Adv..

[9] Wang Y.L., Zhou X.C., Jin Y.H., Zhang X.W., Zhang Z.L., Wang Y., Liu J.L., Wang M., Xia Y., Zhao P. (2019). Strain-dependent Raman analysis of the G* band in graphene. Phys. Rev. B.

[10] Zhang Z.L., Zhang X.W., Wang Y.L., Wang Y., Zhang Y., Xu C., Zou Z.X., Wu Z.H., Xia Y., Zhao P. (2019). Crack propagation and fracture toughness of graphene probed by raman spectroscopy. ACS Nano.

[11] Xie H.M., Han B., Song H.B., Li X.F., Kang Y.L., Zhang Q. (2021). In-situ measurements of electrochemical stress/strain fields and stress analysis during an electrochemical process. J. Mech. Phys. Solids.

[12] Song H.B., Na R., Hong C.Y., Zhang G., Li X.F., Kang Y.L., Zhang Q., Xie H.M. (2022). In situ measurement and mechanism analysis of the lithium storage behavior of graphene electrodes. Carbon.

[13] Xie H.M., Zhang Q., Song H.B., Shi B.Q., Kang Y.L. (2017). Modeling and in situ characterization of lithiation-induced stress in electrodes during the coupled mechano-electro-chemical process. J. Power Sources.

[14] Xie H.M., Song H.B., Guo J.G., Kang Y.L., Yang W., Zhang Q. (2019). In situ measurement of rate-dependent strain/stress evolution and mechanism exploration in graphene electrodes during electrochemical process. Carbon.

[15] Xie H.M., Kang Y.L., Song H.B., Zhang Q. (2019). Real-time measurements and experimental analysis of material softening and total stresses of Si-composite electrode. J. Power Sources.

[16] Adu K.W., Xiong Q., Gutierrez H.R., Chen G., Eklund P.C. (2006). Raman scattering as a probe of phonon confinement and surface optical modes in semiconducting nanowires. Appl. Phys. A.

[17] Ma L.L., Fan X.J., Qiu W. (2019). Polarized Raman spectroscopy-stress relationship considering shear stress effect. Opt. Lett..

[18] Ma L.L., Xing H.D., Li Q., Wang J.S., Qiu W. (2018). Raman stress measurement of crystalline silicon desensitizes shear stress: Only on {001} crystal plane. Jpn. J. Appl. Phys..

[19] Loechelt G.H., Cave N.G., Menéndez J. (1999). Polarized off-axis Raman spectroscopy: A technique for measuring stress tensors in semiconductors. J. App. Phys..

[20] Fu D.H., He X.Y., Ma L.L., Xing H.D., Meng T., Chang Y., Qiu W. (2020). The 2-axis stress component decoupling of {100} c-Si by using oblique backscattering micro-Raman spectroscopy. Sci. China Phys. Mech..

[21] Ma L.L., Xing H.D., Ding Q., Han Y.T., Li Q., Qiu W. (2019). Analysis of residual stress around a Berkovich nano-indentation by micro-Raman spectroscopy. AIP Adv..

[22] Miyatake T., Pezzotti G. (2011). Tensor-resolved stress analysis in silicon MEMS device by polarized Raman spectroscopy. Phys. Status Solidi A.

[23] De Wolf I. High-resolution stress and temperature measurements in semiconductor devices using micro-Raman spectroscopy. Proceedings of the International Symposium on Photonics and Applications.

[24] Lee C.J., Pezzotti G., Okui Y., Nishino S. (2004). Raman microprobe mapping of residual microstresses in 3C-SiC film epitaxial lateral grown on patterned Si (1 1 1). Appl. Surf. Sci..

[25] Kosemura D., Ogura A. (2010). Transverse-optical phonons excited in Si using a high-numerical-aperture lens. Appl. Phys. Lett..

[26] Brunner K., Abstereiter G., Kolbesen B.O., Meul H.W. (1989). Strain at Si-SiO_2_ interfaces studied by Micron-Raman spectroscopy. Appl. Surf. Sci..

[27] Büchler A., Beinert A., Kluska S., Haueisen V., Romer P., Heinz F.D., Glatthaar M., Schubert M.C. (2017). Enabling stress determination on alkaline textured silicon using Raman spectroscopy. Energy Procedia.

[28] Ossikovski R., Nguyen Q., Picardi G., Schreiber J., Morin P. (2008). Theory and experiment of large numerical aperture objective Raman microscopy: Application to the stress-tensor determination in strained cubic materials. J. Raman Spectrosc..

[29] Poborchii V., Tada T., Kanayama T. (2010). Observation of the forbidden doublet optical phonon in Raman spectra of strained Si for stress analysis. Appl. Phys. Lett..

[30] Qiu Z., Im J., Huang R., Ho P.S. Extension of micro-Raman spectroscopy for full-component stress characterization of TSV structures. Proceedings of the IEEE 63rd Electronic Components and Technology Conference.

[31] Becker M., Scheel H., Christiansen S., Strunk H.P. (2007). Grain orientation, texture, and internal stress optically evaluated by micro-Raman spectroscopy. J. Appl. Phys..

[32] Chang Y., He S.S., Sun M.Y., Xiao A.X., Zhao J.X., Ma L.L., Qiu W. (2021). Angle-Resolved Intensity of In-Axis/Off-Axis Polarized Micro-Raman Spectroscopy for Monocrystalline Silicon. J. Spectrosc..

[33] Chang Y., Xiao A.X., Li R.B., Wang M.J., He S.S., Sun M.Y., Wang L.Z., Qu C.Y., Qiu W. (2021). Angle-Resolved Intensity of Polarized Micro-Raman Spectroscopy for 4H-SiC. Crystals.

[34] Gouadec G., Colomban P. (2007). Raman Spectroscopy of nanomaterials: How spectra relate to disorder, particle size and mechanical properties. Prog. Cryst. Growth Charact. Mater..

[35] Gouadec G., Colomban P., Bansal N.P. (2001). Raman Study of Hi-Nicalon-Fiber-Reinforced Celsian Composites: II, Residual Stress in Fiberss. J. Am. Ceram. Soc..

[36] Ma L.L., Zheng J.X., Fan X.J., Qiu W. (2021). Determination of stress components in a complex stress condition using micro-Raman spectroscopy. Opt. Express.

[37] Qiu W., Ma L.L., Li Q., Xing H.D., Cheng C.L., Huang G.Y. (2018). A general metrology of stress on crystalline silicon with random crystal plane by using micro-Raman spectroscopy. Acta Mech. Sin..

[38] Qiu W., He S.S., Chang Y., Ma L.L., Qu C.Y. (2022). Error Analysis for Stress Component Characterization Based on Polarized Raman Spectroscopy. Exp. Mech..

